# Anatomical and Physiological Observations Made upon Decapitated Subjects

**Published:** 1869-07-01

**Authors:** Ch. Robin

**Affiliations:** From Robin’s Journal de Physiol, etc.


### CHICAGO MEDICAL JOURNAL.

Vol. XXVI.—JULY 1, 1869.—No. 13.

### TRANSLATIONS.

### *Anatomical and Physiological Observations made upon Decapitated Subjects.*

BY M. C. ROBIN.

*From Robin's Journal de Physiol, etc.*

I—The Condition of the Cervical and Cephalic Veins.

When the cervical and cephalic veins of decapitated subjects are examined several facts are observed which possess a certain degree of interest in a physiological point of view. Upon the cut surface there may be seen veins clearly cut whose walls are there more or less depressed. The corresponding arteries have their cut extremities retracted to the depth of about a centimetre into the thickness of the tissues.

But what deserves especially to attract the attention of observers is this: that the internal jugular vein incompletely retracted upon itself has its cavity full of air. This replaced the blood at the moment of the abrupt pouring out of the liquid; the air remains there, as is revealed by dissection, by reason of the habitual state of tension of the cervical aponeurosis due to its insertion, and which maintains the veins of the neck in a state of distension above the os hyoides. This has been noticed especially by M. Malgaigne (*Anatomic Chirurgicale*, Paris, 8vo, 1859, 2d edit: t. II., p. 128), in discussing the anatomical observations upon this subject made by M. Bérard (1830), De Grusse (Thése 1849), Richet, etc. Dissection shows, sometimes, also, a little air, in bubbles, mingled with the blood in the trunks of the principal branches which empty into the internal jugular, such as the facial and temporo maxillary veins. The same also sometimes occurs in the veins which surround the transverse apophyses of the first cervical vertebræ, and communicate with the *internal jugular* by means of the inosculating branch which returns from the last to the superior part of the *posterior jugular*.

The most constant fact actually to be noticed consists in the presence of air in the fossa of the jugular vein, in the lateral and other sinuses of the base of the cranium, as well as in the two longitudinal sinuses.

From thence large bubbles of air may be followed in the veins of the cerebral and cerebellar pia mater, and even into several of their subdivisions, along the fissures separating the convolutions.

In the last subject examined I discovered, one hour after death, large bubbles of air in the right sinus, as far as the vena Galien, and in the two large choroid branches. I have never seen them in the veins of the ventricular walls, nor in those which plunge into the gray and white cerebral substance, whatever their volume may have been.

Thus air takes the place of blood wherever the anatomical arrangements are such that the organs of the surface of the body cannot be depressed upon the veins in proportion as the liquid escapes from the vessels abruptly opened. In place of blood, which, in bodies of subjects dead from disease, fills, more or less fully, the vessels, air, alone or mingled with blood, is found under the conditions of death examined here. As to the smaller intra-cranial vessels, other than those referred to, they remain (as in dead bodies generally) more full of blood relatively than those in the extra-cranial regions, the incompressibility of the cranial case, in reference to atmospheric pressure, opposing an obstacle to their retraction, and consequently to the expulsion of the blood in the large venous trunks. This fact is observed, as is well known, even in men and in animals dead from hæmorrhage in consequence of arterial wounds of the limbs.

In this last relation especially, the intra-cranial vessels, other than the sinuses, are not, in decapitated subjects, removed from the influence of atmospheric pressure, as in individuals dead from disease. There is found, indeed, in the first, air, in the place which was occupied by the cephalo-rachidian liquid around the spinal marrow, in the posterior sub-arachnoidean space, and more or less in the spaces at the base of the cranium.

Finally, it must be added, to conclude what refers to the condition of the veins, that the trunk of the vertebral vein adherent to surrounding parts, is slightly retracted upon itself and contains air.

I recall very clearly that my attention was attracted to the presence of air in the cranial sinuses and in the veins of the pia-mater, in 1844 and 1845 by MM. P. Bérard and Denonvilliers. Since then I have verified these facts every time I have had occasion to dissect decapitated persons, and three times in particular, during the last two years, I have vainly sought their indications in treatises of anatomy and physiology. It has been equally impossible for me to discover where Bérard (*Eléments de Physiologie*, Paris, 1858, 2d Edit., t. II, p. 346) has drawn the elements of the following phrase, the only one alluding to the subject under discussion which has come within my knowledge: "Examine," says Bérard, "the head of a decapitated subject, as Beclard, Abercrombie and Professor Bérard have done, or even yet examine the cranial cavity of a person dead of hæmorrhage and you will find there, always, a large quantity of blood."

It is perfectly true that the intra-cranial venous vessels of subjects decapitated, are not absolutely drained of blood. There remains some blood in the smaller veins of the pia-mater, equally in those penetrating the gray and the white substance. But this quantity is sensibly less than in the majority of subjects, dead of different diseases and more or less emaciated, destined for dissection. Moreover, as we have just seen, the cranial sinuses are full of air, or contain more air than blood; the same is true of several of the veins which empty into them, and many of the branches of these last contain bubbles of air, more or less numerous, mingled with blood, filling them for a certain length, and separated from each other by little columns of blood.

### II—The Conditions of the Cervical and Cephalic Arteries—Physiological Consequences of this Condition.

In connection with MM. Legros and Goujon we have observed, in two decapitated subjects, another series of facts more unexpected than the preceding, concerning the presence of air in the vessels. We saw air mingled with blood in the arteries of the head, in the cerebral and cerebellar arteries as far as the arterioles of the pia-mater. M. Legros particularly, investigated these facts with the greatest care in reference to the experiments which he had made in connection with M. Omimus, upon arterial circulation (See *Journal of Anat. and Physiol.*, 1868, p. 371) upon the head of a decapitated person whom I dissected two hours after execution, in September last, with especial reference to the study of the state of the vessels. I determined that the internal and external carotid arteries, incompletely retracted upon themselves, contained frothy blood in which the volume of air surpassed, considerably, that of the blood. All the branches of the arterial hexagon (circle of Willis) of the base of the cranium, very sensibly retracted upon themselves, contained blood mingled with a certain number of air bubbles. The origin of the ophthalmic artery was in the same condition. The arteries originating from the anterior portion and from behind the hexagon were considerably retracted upon themselves without being completely emptied of blood, and their trunks had the appearance of little brownish resistant cords about a millimetre and a half in thickness.

Upon the two other decapitated subjects which I had examined previously with MM. Legros and Goujon, the arteries springing from the hexagon were a little less retracted, and contained bubbles of air, at least equal in quantity to that of the blood with which it was mingled.

The trunks of the principal branches of the external carotid contained blood mingled with bubbles of air. This fact was very striking in the maxillary, and especially in the middle meningeal artery, whose branches contained bubbles of air almost up to the middle of the sides of the cranial arch.

Section of the vertebral arteries made nearly at the level of the transverse apophysis of the fourth cervical vertebræ showed at each side an open orifice alongside of that of the vein: the artery incompletely retracted upon itself in consequence of its adhesion to surrounding organs, contained more air than blood nearly up to the basilar trunk. The latter, much more retracted upon itself than the preceding arteries, contained, like the branches of the hexagon, little columns of blood separated by bubbles of air. The same was true of the cerebellar arteries, but only at their origin.

It is perfectly easy to comprehend under what point of view these facts are worthy of interest.

All physiologists know the remarkable experiments proposed by Legallois, and so well executed for the first time by Brown-Sequard (*Journal de la Physiologie*, Paris, 1858, 8vo, p. 119), which is thus described:

"I decapitated a dog, *taking care to make the section below the point where the vertebral arteries penetrate the osseous canal.* Eight minutes after, pinching of the skin being without effect, I applied a galvanic current of very considerable intensity, to the exposed medulla oblongata, avoiding carefully the passage of the current through the adjacent part. He manifested no movement. The conductors applied to the protuberence could produce no effect. Ten minutes after the cessation of the respiratory movements of the nostrils, of the lips and of the lower jaw, I fitted to the four arterial trunks of the head canulæ which were connected by tubes of caoutchouc with a brass cylinder, by means of which I injected blood charged with oxygen by the aid of a syringe. In two or three minutes, after some slight disorderly movements, I detected movements of the eyes and of muscles of the face which seemed to be directed by the will. I prolonged the experiment a quarter of an hour, and during all that period these movements, apparently voluntary, continued to take place. After having discontinued the injection these movements ceased, and were soon replaced by convulsions of the eyes and of the face, by respiratory movements of the nostrils, of the lips and of the jaws, and, finally, by the tremors of the death-struggle. The pupil dilated and contracted as in ordinary death.

This experiment, adds Brown-Sequard, demonstrates positively the possibility of the return of the vital properties and of the functions of the encephalon under the influence of blood charged with oxygen."

There can be no doubt that this experiment, made upon man under conditions analogous to those in which Brown-Sequard was placed, would give results of the same order, as to the phenomena of central innervation, and of motor excitation in particular.*

It is very certain that movements of the lids and lips may be determined under these conditions, like the return of the nervous tissue to reflex actions, if not to voluntary movements, by tickling or pinching the skin at this or that point of the face; movements obtained in an analogous manner to that which Brown-Sequard (Comptes rendu de l'Academie des Sciences, 1851, t. XXXII, p. 856), has perceived whilst making experiments of this kind upon the posterior portion of the bodies of rabbits.

But it will be seen after what has been already said of the presence of air in the vessels of the neck and of the cranial cavity, that contrary to what some physiologists have thought from the indications of M. Velpeau (Leçons sur la physiologie du System Nervoux, Paris, 1866, 8vo., p. 460,) this experiment could not be made upon the head of a decapitated person. It could not succeed, or at least furnish conclusive results, as upon the body of a man killed by balls striking below the neck. Under such circumstances only, which it would not be impossible to meet, section of

*It may be added that this experiment, and others likewise, which Brown-Sequard has described in this same work, demonstrate the functional anatomy of the central and peripheral nervous cells and tubes, in the same manner as that of other species of anatomical elements whose properties are manifested while the characteristic muscular condition of the state of organization persist, and the relations of the element with the blood permit its incessant molecular renovation. It is sufficient that these relations cease, in order that the elements should lose their properties, and these which would not return, if this state were maintained, would re-appear just in so far as these relations were re-established, before the chemical decomposition of this or that principle in the substance of the element may have taken place. Then, whilst it is yet too slight to modify the form or the structure of the elements, this decomposition is, nevertheless, sufficient already to destroy the existence of the particular molecular state which is essential to the state of organization: in order that consequently the conditions of this continuous renovation should no longer exist; aside from the accomplishment of which no other property of the organic series can manifest itself.

the neck might be performed, *below the level at which the vertebral arteries penetrate their canal*, conditions of which Brown-Sequard has not misunderstood the importance.

It is indispensable indeed to divide the arteries at one point, and even to effect it in such a manner that the retraction of their walls may be accomplished in proportion as the blood pours out, so that this last may not be partially filled with air as occurs in the case of guillotined subjects, through the vertebrals.

It is likewise so with the carotids which, instantaneously and simultaneously cut transversely and entirely, lose immediately their blood under conditions which are no where met with outside of this mode of capital execution.

Now for the vertebrals this retraction is not possible, nor at least is not accomplished except after a prolonged traction exercised upon the surrounding layers of tissue throughout their whole extent, in the foramina at the base of the transverse cervical apophyses. Cut at one point of this place, they invariably permit the air to enter in place of the blood which escapes, and by reason of the incompressibility of the cranial vault, this air accumulates thence in the intra-cerebral arteries which empty themselves more or less in different individuals.

Although the conditions are not the same in this relation for the carotids, it is however certain that, in decapitated subjects they are not retracted upon themselves up to the point of complete obliteration, and that they contain air which extends into these or those branches as we have already seen.

Hence the impossibility of making into the head of a decapitated person, a few minutes after death, an injection capable of giving results such as those that M. Brown-Sequard has obtained under the conditions which he has noted.

It is well known indeed that in every injection made into the arteries or veins, one of the essential precautions to be taken is to prevent the entrance of air before the column of liquid is introduced. Otherwise, as occurs both in the living and the dead, the presence of bubbles or of a column of air is seen in the little vessels or in the capillaries opposing an obstacle to the progression of the liquid, and soon the continuation of the pressure induces ruptures of the tubes with foci of extravasation, or with infiltration of the fluid into the elements of the surrounding tissues. Hence artificial apoplectic extravasations going in this case counter to the intention of the experiment; for the result would not be certainly comparable to those which experiments made upon dogs have furnished, and from thence one would be led to conclude dissimilarities which do not exist between the fundamental acts of innervation. The results might even be negative, without permitting the deduction of any logical conclusion therefrom.

Thus the circumstances in which the vessels are cut in decapitated subjects are such that the air takes the place of the blood which jets out; and that, too, not only in the veins but likewise in the arteries into which blood ought to be forced in order to replace the nervous elements in conditions of internal constitution such that their extinct properties may reappear.

The conditions which render the experiment valuable and conclusive fail then here, and those which exist constitute in this particular case difficulties impossible to surmount in order to attain the desired result. This is moreover but one more illustration among those which show how often, in the conception and in the execution of every experiment, it is necessary to take into consideration the conditions of accomplishment of the phenomenon which one endeavors to modify in order to analyze it more clearly, and how, in this respect, no intellectual superiority can be substituted for the observance of these conditions of organic, chemical and physical order.

It may be considered, however, as certain that the phenomena of central innervation placed in evidence by the movements observed in the dog during the experiment of M. Brown-Sequard, and those which could be established in man by placing him in similar conditions could not be regarded as associated and co-ordinated into thoughts becoming motors of the voluntary execution of this or that movement.

For those who have studied, as have physiologists and physicians, the conditions, as well intrinsic as extrinsic, necessary to the regularity of intellectual acts, the solitary fact of the absence of the cephalo-rachidian liquid in the head separated from the trunk, suffices independently of questions of pressure and of the regularity of the course of the blood injected, to lead to the belief that the acts of cerebral innervation which are manifested then, are hardly comparable but to those of which men do not preserve the recollection, and which take place, for example, at the beginning of the cessation of a syncope, as also in delirium, during an access of fever, etc.

[CONTINUED IN NEXT NUMBER.]

